# Supplementary material for: New Variants of Pseudomonas aeruginosa High-Risk Clone ST233 Associated with an Outbreak in a Mexican Paediatric Hospital
Source: Microorganisms. 2022 Jul 29;10(8):1533. doi: 10.3390/microorganisms10081533 (PMC9414371; doi:10.3390/microorganisms10081533)
Supplement: Supplementary file 1 [file microorganisms-10-01533-s001.zip › Supplementary Figure S1.pdf]

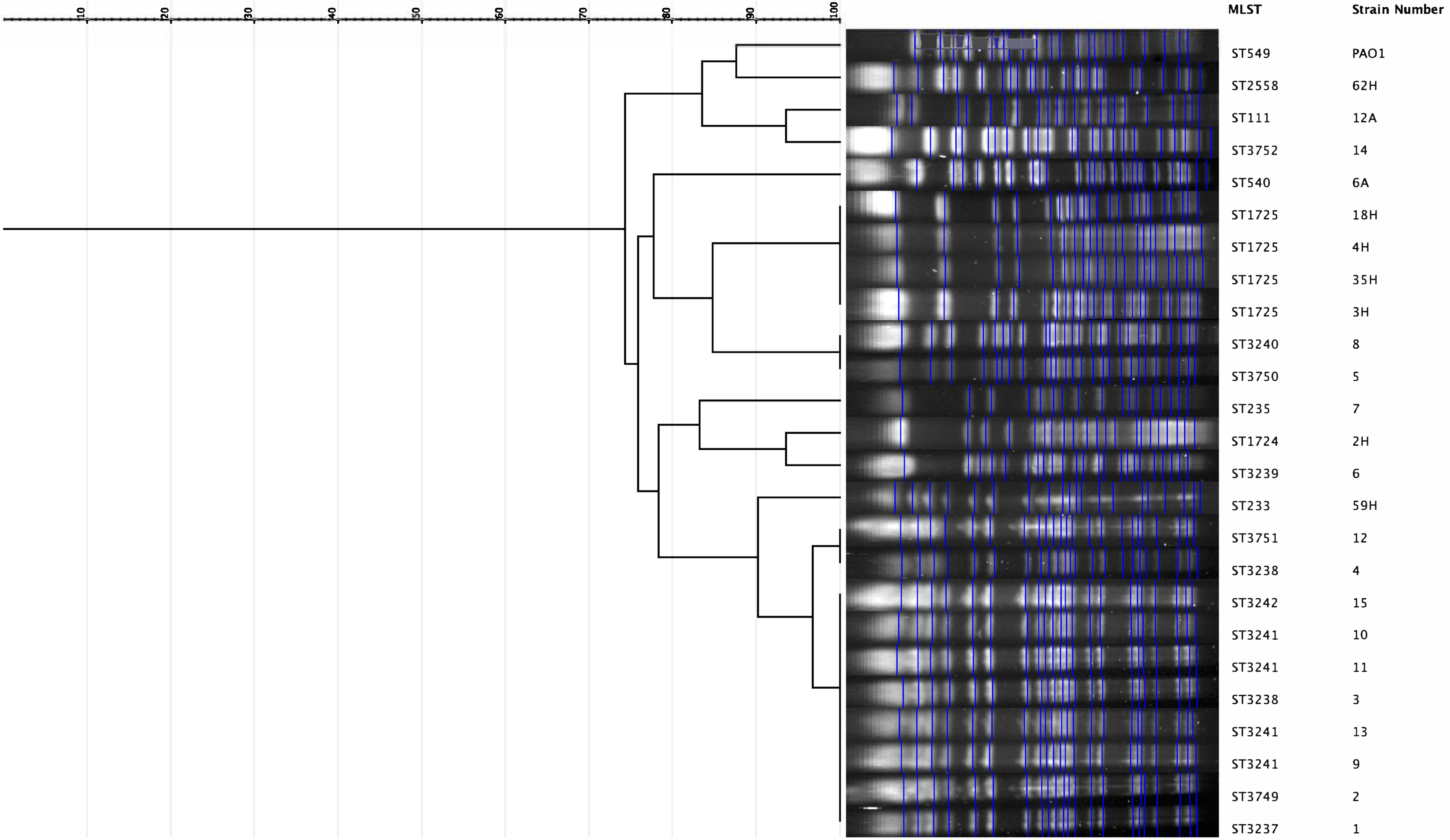

Supplementary Figure S1. Phylogenetic tree of *P. aeruginosa* strains isolated in the HIMFG based on their electrophoretic pattern (PFGE)

Electrophoretic patterns were obtained by PFGE with the *Spe* I enzyme. The phylogenetic tree was built with the Unweighted Pair Group Method with Arithmetic Mean (UPGMA) according to the PFGE electrophoretic patterns and using the gelJv2 program. Ten previously isolated and reported STs in the HIMFG, and the strains reported in this study (n=15) are incorporated. ST and strain number are also shown.
