# Supplementary material for: New Variants of Pseudomonas aeruginosa High-Risk Clone ST233 Associated with an Outbreak in a Mexican Paediatric Hospital
Source: Microorganisms. 2022 Jul 29;10(8):1533. doi: 10.3390/microorganisms10081533 (PMC9414371; doi:10.3390/microorganisms10081533)
Supplement: Supplementary file 1 [file microorganisms-10-01533-s001.zip › Supplementary Table S1.pdf]

Supplementary Table S1. Clinical patients features

Patient	Strain	Infection	Hospital Ward	Diagnosis	Age	Source	Isolation date	Risk factors	Antibiotics	Symptoms
	12	Multi-dose meropenem vial	NICU			J18M790	22/06/18			
P 1	4		STx		9 years	Blood culture	26/06/18			
	13	Central line-associated bloodstream infection	STx	Craniopharyngioma<br>Ventriculitis<br>Surgial wound dehiscence<br>PO CSF pseudocyst	9 years	Blood culture	23/06/18	Central venous catheter<br>Previous surgeries<br>Previous antibiotics	Cefepime<br>Vancomicin<br>Meropenem<br>Colistin	Fever<br>Tachycardia
P 2	11		Emergency Oncology		15 years	Blood culture	16/06/18			
	10		Emergency Oncology		15 years	Blood culture	16/06/18			
	3	Fever and neutropenia	Emergency Oncology	Non-metastatic inguinal Ewing Sarcoma<br>Obesity	15 years	Blood culture	16/06/18	Chemoterapy June 4 to 8 vincristine, doxorrubicin and cyclophosphamide<br>Private physician applied single dose: ceftriaxone,<br>cefixime and gentamicin<br>Last hospitalization April 23 to 27 due to fever and neutropenia in Oncology	Cefepime<br>Amikacin	Odynophagia<br>Otalgia<br>Fever
P 3	9	Central line-associated bloodstream infection	Emergency	Acute lymphoblastic leukemia<br>Septic shock	10 years	Blood culture	14/06/18	Port catheter (6 months)<br>Chemotherapy	Meropenem<br>Vancomicin	Hypothermia<br>Tachycardia<br>Hypotension
P 4	1	Bloodstream infection	Emergency Surgery	Lennox Gastaut Syndrome<br>Altered swallowing mechanics	3 years	Blood culture	09/06/18	Hospitalization<br>Peripheral venous line<br>O2 per mask<br>Previous Antibiotics	Meropenem<br>Amikacin	Fever<br>Hypothermia<br>Tachycardia<br>Respiratory Distress

**P 5**	**2**	Bloodstream infection	Emergency	Right scapular rhabdomyosarcoma with lung metastases. Post-operated wide local resection	3 years	Blood culture	03/06/18	Peripherally inserted central venous catheter (PICC)<br>Outpatient chemo procedures	Cefepime<br>Amikacin<br>Colistin<br>Rifampicin	Fever<br>Tachycardia<br>Cough
**P 6**	**15**	Urinary tract infection related to urinary catheter	NICU<br>Surgery	Intestinal Neurogliomatosis l type II	1 year 9 months	Urine culture	25/06/18	Cystostomy Previous antibiotic (ertapenem)	Meropenem	Fever<br>Tachycardia<br>Increased cost due ileostomy<br>Dehydration
**P 7**	**8**	Bloodstream infection	NICU	Anorectal malformation with vestibular fistula<br>Right Hydronephrosis<br>Post-intubatipn subglottic stenosis	3 months	Urine culture	06/06/18	Central venous catheter<br>Stay in Neonatology<br>Previous Antibiotics<br>Dysfunctional catheter	Meropenem<br>Vancomicin	Fever<br>Tachycardia<br>Respiratory Distress
**P 8**	**5**	Bloodstream infection	NICU	Perinatal asphyxia<br>Hydronephrosis	1 month	Blood culture	03/06/18	Central venous Catheter<br>Previous Antibiotics (meropenem, vancomicyn)	Piperacilin-Tazobactam<br>Amikacin<br>Colistin<br>Cefepime	Fever<br>Tachycardia
**P 9**	**14**	Fever and neutropenia Hospitalized from June 29 to July 4<br><br>Viral Panel + Rhinovirus	Emergency<br>Oncology	Primary Neuroblastoma of the left adrenal gland. Stage 4 (bone, bone marrow and skin metastasis) with high risk in neoadjuvant.	5 months	Blood culture	03/07/18	Hospitalized in internal medicine from April 27 to May 5 due to sepsis<br>Tx cefepime 10 days<br>Chemoterapy May 10 to 15, June 13 to 17 cyclophosphamide, cisplatin, doxorubicin<br>Percutaneous left axillary catheter on 14/06/18	Cefepime<br>amikacin	Fever<br>Runny nose<br>Tachycardia<br>Cough

**P 10**	**7**	Urinary tract infection	Infectious diseases outpatient	Left hydronephrosis PO percutaneous extracorporeal nephrectomy Renal litiasis (pb colonization of 2 l residual stones in left renal pelvis.	14 years	Urine culture	15/06/18	Previous hospitalization 30/03/18 to 14/04/18 in urology due to complicated recurrent UTI with isolated multidrug resistant *P. aeruginosa* treated with colistin, ciprofloxacin 14 days Mar 16 to 19 hospitalizated due to percutaneus laser nephrolithotomy and double J catheter, nephrostomy catheter and urethral catheter were placed.	Fosfomicin Nitrofurantoin prophylaxis	No
	**6**		Infectious diseases outpatient		14 years	Urine culture	01/06/18			

Patient: number of patient; Strain: number of the strain isolated from the patient; Hospital Ward: Emergency (yellow), Surgical Therapy (STx, blue), Oncology (pink), Neonatal Intensive Care Unit (NICU, purple), Infectious diseases outpatient (green). Clade II/CC233 (blue); Clade I/CC233 (yellow); Clade III/CC111 (purple); Clade IV/CC235 (green).
