# Supplementary material for: New Variants of Pseudomonas aeruginosa High-Risk Clone ST233 Associated with an Outbreak in a Mexican Paediatric Hospital
Source: Microorganisms. 2022 Jul 29;10(8):1533. doi: 10.3390/microorganisms10081533 (PMC9414371; doi:10.3390/microorganisms10081533)
Supplement: Supplementary file 1 [file microorganisms-10-01533-s001.zip › Supplementary Table S2.pdf]

Supplementary Table S2. Accession numbers (ID) of the *P. aeruginosa* strains analysed in this work

Id at PubMLST.org	Isolate	ST
6714	HIM1/18	3237
7863	HIM2/18	3749
7898	HIM3B/18	3238
6715	HIM4/18	3238
7864	HIM5/18	3750
6716	HIM6/18	3239
7899	HIM7/18	235
6717	HIM8/18	3240
6718	HIM9/18	3241
7900	HIM10/18	3241
7901	HIM11/18	3241
7865	HIM12/18	3751
7902	HIM13/18	3241
7866	HIM14/18	3752
6719	HIM15/18	3242

IDs available at the public database for molecular typing: PubMLST.org.
